# Role of Growth Hormone (GH) and Other Somatotropic Axis Elements in Retinal Neuroprotection

**DOI:** 10.3390/cimb48030296

**Published:** 2026-03-11

**Authors:** David Epardo, Carlos Arámburo, Carlos Guillermo Martínez-Moreno

**Affiliations:** Departamento de Neurobiología Celular y Molecular, Instituto de Neurobiología, Campus Juriquilla, Universidad Nacional Autónoma de México, Querétaro 76230, Mexico; epardodavid@comunidad.unam.mx

**Keywords:** growth hormone, retina, somatotropic axis, neuroprotection

## Abstract

Various pathological conditions can result in retinal degeneration and, in extreme cases, blindness. Unfortunately, current treatments for many of these conditions are not effective, and ongoing research encounters numerous obstacles due to the complex nature of these diseases, which involve multiple simultaneous mechanisms that cannot be controlled by a single factor. Therefore, there is an urgent need to propose and test new molecules that could exert protective effects at multiple levels. Traditionally, growth hormone (GH) has been viewed as a detrimental factor contributing to develop retinopathies. However, recent investigation has debunked this notion, revealing that GH treatment exerts strong neuroprotective effects during retinal injury. It is crucial to recognize that these actions are not exclusive to GH, since other related molecules may also be involved. Therefore, it is important to collect relevant existing evidence regarding GH axis translational research in order to understand its potential as a therapeutic option for retinal degeneration.

## 1. Introduction

Retinal injury and optic nerve damage trigger emergency mechanisms that initiate compensatory and repair processes through a complex network of molecular interactions involving growth factors, neurotrophins, neuropeptides, cytokines, and hormones [1]. A wide range of conditions such as mechanical trauma, infections, aging, metabolic diseases, environmental factors, genetic alterations, or congenital disorders can impair visual function, many of which result in cell death within the retina or along the neuronal visual pathway [2]. Although certain pathologies can be treated with surgery or pharmacological interventions, vision loss is frequently associated with progressive neurodegeneration and is often irreversible. However, it is important to consider that, before extensive structural or physiological damage develops, there is a narrow therapeutic window in which neurotrophic and neuroprotective agents may be used to prevent cell death and irreversible degeneration [3].

Numerous studies have explored the effect of neurotrophins and neuroprotective peptides for treating retinal neurodegenerative diseases [4]. Despite their potential, current neurotrophic therapies face significant limitations, including receptor desensitization due to repeated dosing, secondary adverse effects, and challenges related to optimal delivery (timing, dosage, and tissue targeting), which are inherently complex and involve multiple biological processes [5]. Thus, molecules such as brain-derived neurotrophic factor (BDNF), ciliary neurotrophic factor (CNTF), or neurotrophin-3 (NT-3) have shown anti-apoptotic, homeostatic, and pro-survival effects; however, their clinical application still remains elusive, largely due to their limited receptor distribution and the rapid development of tolerance upon repeated administration [5]. Consequently, to date, no neurotrophic factor-based therapies are currently in routine clinical use [6,7]. Advancing therapeutic strategies will require a deeper understanding of the regulatory mechanisms that modulate the effects of these molecules in the retina, the identification of new candidates capable of exerting multiple beneficial actions simultaneously, and an appreciation of the molecular pathways that are activated during retinal neurodegeneration. Moreover, neural regeneration in mammals is intrinsically limited, and despite the significant advances made in non-mammalian vertebrates, in which multiple regenerative pathways and neuroprotective factors have been successfully studied, their translation to mammals, and especially primates, continues to represent a substantial challenge [8].

Within this context, the somatotropic axis, and particularly growth hormone (GH), has gained increasing attention as a potential protective agent in the retina. The local transcription, translation, and release of GH and some of its upstream and downstream regulatory factors have been documented in ocular tissues and throughout the central nervous system (CNS) in several vertebrates [9,10,11,12,13,14,15,16]. Furthermore, treatments with exogenous GH and interacting molecules have shown protective and regenerative properties in various models of neural injury [17,18,19].

It has been suggested that several elements of the somatotropic axis may hold significant promise for visual system neuroprotection; although many of their interactions, regulatory pathways, and potential synergistic effects in this regard remain to be fully understood [20]. This review compiles and discusses current evidence from basic and translational research that highlights the therapeutic potential of GH and other peptides directly or indirectly involved in the somatotropic axis for preventing, mitigating, and controlling neuronal cell death in retinal degenerative diseases.

To ensure transparency in the selection of the literature discussed, references were identified through systematic searches conducted in PubMed, Scopus, and Google Scholar covering publications from January 1980 to December 2025. The search strategy combined keywords including “growth hormone”, (or every specific axis element discussed), “somatotropic axis”, “retina”, “retinal degeneration”, “optic nerve injury”, “neuroprotection”, “neuroregeneration”, and “central nervous system”. Additional relevant studies were identified through manual screening of reference lists of selected articles. Both experimental and translational studies published in English were considered.

## 2. Single-Cell RNA Sequencing Data

**Figure 1:**
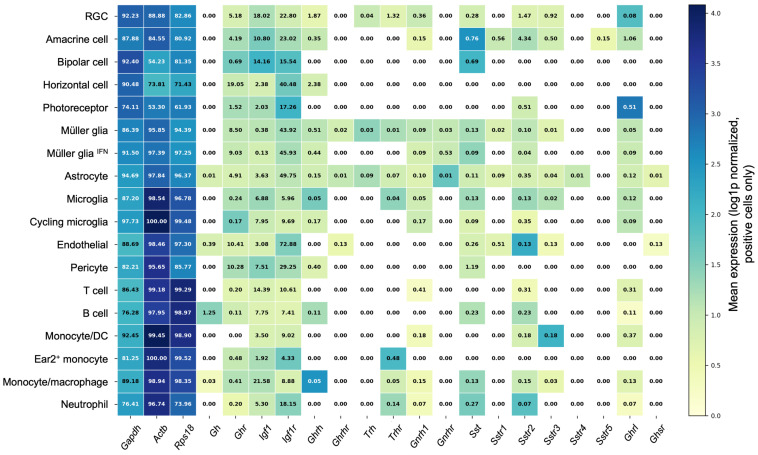
Cell-type-resolved expression of somatotropic-axis and housekeeping genes in the mouse retina. Heatmap shows mean log1p-normalized expression among positive cells (color scale) for each gene (columns) across major retinal cell types (rows); numbers within cells report the percentage of cells in each type with detectable expression (raw UMI count > 0) to two decimals, and white cells indicate 0.00% detection for that group (masked means are therefore not shown to avoid misinterpretation). Data are aggregated from the publicly available single-cell RNA-seq dataset GSE199317. Gene symbols with their full names are: *Gapdh* (glyceraldehyde-3-phosphate dehydrogenase), *Actb* (beta-actin), *Rps18* (ribosomal protein S18), *Gh* (growth hormone), *Ghr* (growth hormone receptor), *Igf1* (insulin-like growth factor 1), *Igf1r* (insulin-like growth factor 1 receptor), *Ghrh* (growth hormone–releasing hormone), *Ghrhr* (growth hormone–releasing hormone receptor), *Trh* (thyrotropin-releasing hormone), *Trhr* (thyrotropin-releasing hormone receptor), *Gnrh1* (gonadotropin-releasing hormone 1), *Gnrhr* (gonadotropin-releasing hormone receptor), *Sst* (somatostatin), *Sstr1* (somatostatin receptor type 1), *Sstr2* (somatostatin receptor type 2), *Sstr3* (somatostatin receptor type 3), *Sstr4* (somatostatin receptor type 4), *Sstr5* (somatostatin receptor type 5), *Ghrl* (ghrelin), and *Ghsr* (growth hormone secretagogue receptor).

Publicly available single-cell RNA sequencing data (GEO accession GSE199317; Nature Immunology) were used for the exploratory expression analysis shown in Figure 1 [21]. The deposited dataset includes uninjured control mice retinas and multiple post-optic nerve crush (ONC) time points (12 h, 1 d, 2 d, 4 d, 1 week, 2 weeks), and the analysis reported here aggregates the annotated cells across those conditions. The raw UMI count matrix (GSE199317_ONC-retina.mtx), cell barcode table (GSE199317_ONC-retina_cell.tsv), gene annotation table (GSE199317_ONC-retina_gene.tsv), and cell-type annotation table (GSE199317_ONC-retina_celltype.tsv) were downloaded from the NCBI Gene Expression Omnibus. The original experimental procedures, animal handling, injury model and sequencing protocols follow those reported in the cited publications and are acknowledged accordingly. Data processing and plotting were performed with a custom Python (version 3.13.0) script (provided as Supplementary Code) using standard open-source packages (Scanpy (version 1.11.4)/AnnData (version 0.12.1), pandas (version 2.3.0), numpy (version 2.2.6), scipy (version 1.16.1), seaborn (version 0.13.2) and matplotlib (version 3.10.3)). Gene and cell barcodes were assigned from the provided TSVs and only cells carrying a “major” cell-type annotation in the deposited metadata were retained; these original annotations were mapped to a curated set of broad cell-type groups (mapping table provided in the Supplementary Materials) and only mapped cells were analyzed. Raw UMI counts were preserved for detection calls (a cell was considered positive for a gene if raw UMI > 0). For magnitude estimates, counts were library-size normalized to 10,000 counts per cell and transformed by the natural logarithm (log1p). We evaluated a focused gene panel composed of housekeeping controls (*Gapdh*, *Actb*, *Rps18*) and somatotropic/neuroendocrine genes discussed in this review (*Gh*, *Ghr*, *Igf1*, *Igf1r*, *Ghrh*, *Ghrhr*, *Ghrl*, *Ghsr*, *Trh*, *Trhr*, *Gnrh1*, *Gnrhr*, *Sst*, *Sstr1, Sstr2*, *Sstr3*, *Sstr4*, *Sstr5*), and only genes present in the deposited matrix were included. For each broad cell type × gene pair we computed the total cell count, the number and percentage of cells with detectable expression (percent positive = 100 × [cells with raw UMI > 0)/total cells in group]; reported to two decimals), and descriptive statistics of log1p-normalized expression both across all cells in the group and restricted to positive cells (mean, median, standard deviation; provided in the Supplementary Material). To avoid misleading interpretation of averaged values when no cells showed detectable expression, any positive-only mean corresponding to 0.00% positive was masked (set to NaN) prior to plotting and saved in the pivot tables; these masked values are rendered as white cells in the heatmap to indicate absence of detected expression rather than a low non-zero mean. All summary metrics, pivot tables and the label-mapping table are supplied as Supplementary Material. The results are exploratory and intended to guide follow-up experimental validation to confirm endogenous, cell-type-specific expression.

## 3. Growth Hormone in the Retina

### 3.1. Retinal GH and GHR

**Figure 2:**
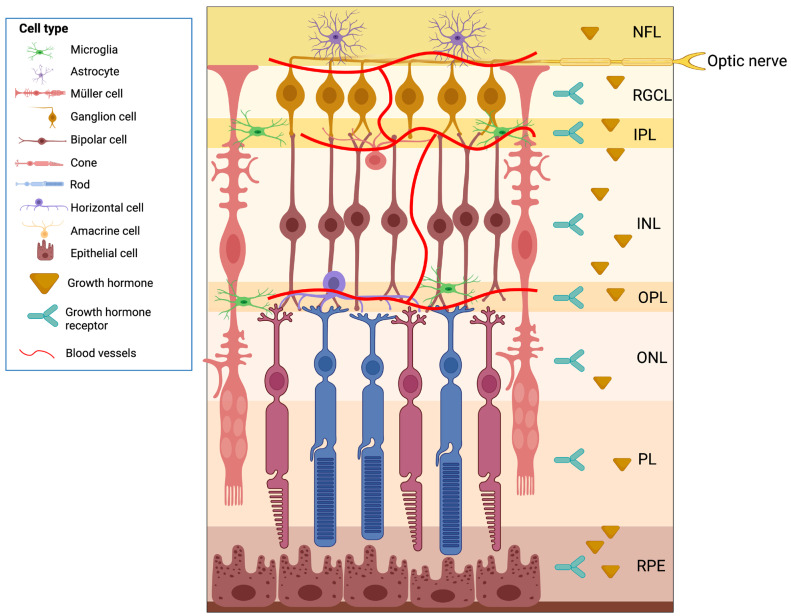
Schematic representation of the distribution of growth hormone (GH) and growth hormone receptor (GHR) across the retinal layers. The diagram illustrates the major retinal cell types, including neurons (ganglion, amacrine, bipolar, horizontal, cone, and rod photoreceptors), macroglia (Müller cells, astrocytes), microglia, epithelial cells, and vascular elements, arranged according to their localization in the nerve fiber layer (NFL), retinal ganglion cell layer (RGCL), inner plexiform layer (IPL), inner nuclear layer (INL), outer plexiform layer (OPL), outer nuclear layer (ONL), photoreceptor layer (PL), and retinal pigment epithelium (RPE). Both GH (yellow triangles) and GHR (blue receptors) have been reported in all retinal layers, consistent with their potential to modulate neuronal, glial, and epithelial cell physiology throughout the retina. Blood vessels are indicated in red. This figure summarizes anatomical evidence supporting the widespread presence of somatotropic axis components within the retina. Created in BioRender. Epardo, D. (2026) https://BioRender.com/cxk6pgs (accessed on 3 March 2026).

The extrapituitary expression of hypophyseal hormones such as GH, prolactin (PRL), and thyroid-stimulating hormone (TSH) in various tissues, originally considered only as targets of their systemic actions, is now well documented and widely accepted [22]. GH and its receptor (GHR) are expressed in the retina across multiple vertebrate classes, including fish, reptiles, birds, and mammals, suggesting a broad spectrum of roles ranging from embryonic development to adulthood [9,10,11,12,13,14,15,16]. Recently, Pérez-Ibave and colleagues [9,10] demonstrated GH and GHR expression in all retinal layers of baboon and human eyes (Figure 2). However, a precise cell-type-specific characterization of GH and GHR expression in the mammalian retina remains lacking. To date, detailed cell-type-specific information on the retinal expression of GH, GHR, and other components of the somatotropic axis in mammals relies primarily on transcriptomic analyses. Publicly available single-cell RNA sequencing datasets allow an indirect and exploratory assessment of their potential local expression across major retinal cell populations; however, low-abundance transcripts are frequently under-detected in single-cell approaches due to technical dropout, which may lead to false-negative expression calls [21]. These datasets provide a useful reference framework that requires targeted experimental validation to confirm endogenous, cell-type-specific expression (Figure 1) [21].

The local expression of both GH and GHR appears to be developmentally regulated in several species. For example, in the chicken retina, GH expression is initially confined to retinal ganglion cells (RGCs) during early development, but becomes more widespread at later stages. This dynamic pattern of expression in different cell types across developmental stages suggests that GH plays multiple roles in regulating retinal physiology and function [23,24,25,26].

**Figure 3:**
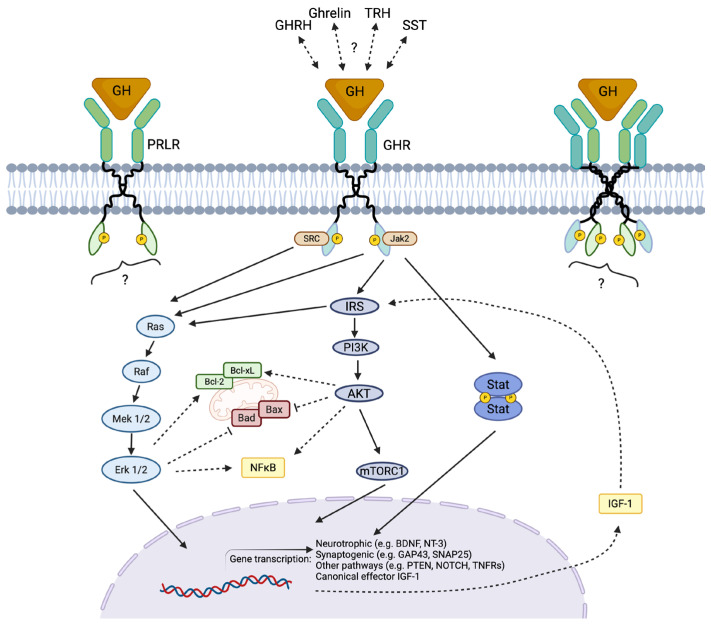
Schematic representation of canonical and putative signaling pathways engaged downstream of growth hormone (GH) in retinal cells. GH binding to GHR homodimers (central), to PRLR homodimers (left) or heteromultimeric receptors (right) promotes receptor conformational change and rapid tyrosine phosphorylation of associated kinases (e.g., JAK2 and SRC), leading to recruitment of adaptor proteins such as insulin receptor substrates (IRS). These proximal events feed into three principal downstream modules: the PI3K → AKT → mTOR pathway, the RAS → RAF → MEK → ERK MAP-kinase cascade, and JAK2-dependent STAT activation, each of which can regulate transcriptional programs and cellular physiology. Downstream consequences relevant to neuroprotection include modulation of BCL-2 family members (Bcl-2, Bcl-xL, BAD, BAX) and inhibition of pro-apoptotic signaling, activation of NF-κB and other survival pathways, and induction of neurotrophic/synaptogenic gene expression (e.g., BDNF, NT-3, GAP43, SNAP25). GH signaling can promote local IGF-1 production (dashed arrow) and other neuroendocrine peptides (GHRH, ghrelin, TRH, SST) may modulate GH/GHR activity or act in parallel. Solid arrows indicate established activation steps; dashed arrows and question marks denote indirect, hypothesized or cell-type–dependent links. Created in BioRender. Epardo, D. (2026) https://BioRender.com/y24x9ex (accessed on 3 March 2026).

GH exerts its biological effects primarily by binding to GHR and inducing conformational changes in the GHR homodimer, a mechanism conserved in nearly all known GH-responsive cells [27]. This structural rearrangement activates canonical intracellular pathways, particularly the JAK2/STAT signaling cascade. In the retina, GHR activation predominantly leads to STAT5 phosphorylation [27,28], although additional pathways such as Notch, AKT, and MAPK are also concurrently activated [29] (Figure 3). The onset of JAK/STAT signaling triggers the transcription of a wide range of downstream target genes involved in cell survival, proliferation, differentiation, and neuroprotection [30].

Beyond STAT5, GH signaling has been shown in other cellular contexts (e.g., 3T3-F442A fibroblasts and Chinese hamster ovary cells) to activate STAT3, STAT5a, and STAT5b, leading to the nuclear accumulation of both homodimeric and heterodimeric forms of STATs. This complexity helps explain the diverse, tissue or cell-dependent effects of GH, including its variable roles in the central nervous system (CNS) following injury [17,18,19,31,32,33,34].

### 3.2. Retinal Actions of GH

Upon binding to its receptor (GHR) in the retina, GH activates multiple signaling pathways, and the ligand–receptor complex undergoes internalization via the endosomal system. Once internalized, it travels within the cytoplasm and accumulates near the nucleus, where it can translocate through the importin α/β (IMPα/β) system and modulate transcriptional processes as well as chromatin remodeling [35,36,37]. This nuclear translocation adds an additional layer of complexity to GH’s pleiotropic functions. Importantly, GH is capable of permeating the blood–brain barrier, as demonstrated in both mammals and birds, where it has been detected in several regions of the CNS following systemic administration [38,39,40]. In the retina, both intravitreal and systemic injections of Cy3-labeled GH have shown uptake by RGCs in chicken embryos [39], and a receptor-mediated mechanism of this uptake has been demonstrated in quail neuroretinal-derived (QNR/D) cells [39,41]. Thus, GH actions in the retina may arise from both local synthesis and systemic sources.

GH activates several downstream signaling cascades through its interaction with intracellular mediators. These include the canonical JAK2/STAT pathway, the mitogen-activated protein kinases (MAPK) cascade, the phosphatidylinositol-3-kinase (PI3K)/Akt pathway via insulin receptor substrates (IRS), the signal regulatory protein α (SIRPα/SHPS1) which recruits SHP2 phosphatase, and the SH2B1 scaffold protein, which modulates the actin cytoskeleton by enhancing JAK2 activation [27,42,43,44]. In the retina, the JAK/STAT, MAPK, and PI3K/Akt pathways are primarily implicated in mediating GH’s effects [19,29,45] (Figure 3).

Through these pathways, GH exerts neuroprotective actions that include promoting cell survival, reducing apoptosis, supporting axonal growth, and enhancing synaptogenesis. In chicken neuroretinal cultures, GH restricts apoptosis via PI3K/Akt activation, accompanied by increased expression of the anti-apoptotic protein Bcl-2 [18]. In human postmortem retinas, GH immunoreactivity negatively correlated with apoptotic markers and positively correlated with healthy RGCs [46]. These effects are often associated with PI3K/Akt activation and enhanced phosphorylation of STAT5 and ERK1/2 [18,41]. While exogenous GH can activate JAK/STAT, MAPK, and PI3K/Akt cascades, the latter two are most strongly linked to its anti-apoptotic and neuroprotective roles, as Akt promotes cell survival, whereas MAPK is strongly associated with neuroprotection in the retina [19,47,48,49,50,51] (Figure 3).

Notably, GH-mediated neuroprotection is not limited to in vitro systems or embryonic stages. In vivo, chronic intravitreal GH administration in chickens exposed to excitotoxic injury induced with kainic acid (KA) promoted retinal cell proliferation, preserved retinal layer structure, and increased survival and synaptogenic markers [19,29]. Also, in rats subjected to optic nerve crush (ONC), systemic administration of a growth hormone releasing hormone (GHRH) agonist elevated intraocular GH levels, promoted RGC survival, and potentiated the neuroprotective effects of lens injury and zymosan [47]. More recently, systemic GH administration after ONC demonstrated both acute (24 h) and chronic (14 days) protective effects, including preservation of RGCs, optic nerve integrity, and regulation of Bcl-2 family proteins toward an anti-apoptotic environment, especially in the RGC layer [17]. Functional recovery was evidenced by full-field electroretinogram (ERG), where GH prevented reductions in B-wave and oscillatory potentials (OPs) under scotopic conditions and mitigated their latency delays. Subsequent studies also reported GH-mediated attenuation of retinal neuroinflammation and partial preservation of the optomotor reflex [52].

GH also modulates additional signaling pathways in the retina. For example, it can indirectly enhance Notch signaling via STAT5 binding sites in Notch1 and Notch2 promoters [29] and can shift tumor necrosis factor (TNF) receptor balance by downregulating TNFR1 and upregulating TNFR2 [20,29]. Moreover, GH downregulates PTEN, thereby enhancing PI3K/Akt activation [29]. These observations suggest that many GH actions arise from secondary regulation of interacting molecules rather than direct receptor activation alone. A more comprehensive understanding of GH signaling is therefore required to fully elucidate its role during retinal protection.

Another important action described for GH in the retina is its ability to promote axonal growth and synaptogenesis, processes that are particularly relevant because they may slow or halt the progression of axonopathies before they trigger neuronal death and progressive neural damage [4,17,19,41]. The restoration of damaged neural networks in the retina and visual processing areas remains an active field of research, although the specific contributions of GH in this context have not yet been extensively explored [9]. Nevertheless, available evidence indicates that GH treatments can induce synaptic neuroprotection, axonal growth, and even synaptic regeneration [17,19,41]. These effects have been demonstrated both in vitro and in vivo. In primary chicken neuroretinal cell cultures treated with physiological concentrations of exogenous GH, an increase in average neurite length was observed [18]. When co-incubated with KA, GH not only increased neurite length but also enhanced the number of long neurites, suggesting a protective and/or regenerative effect, although the study did not determine whether GH specifically promoted neurite regrowth or primarily exerted neuroprotection [18]. Furthermore, GH has been shown to upregulate synaptogenic and axogenic factors, including Synaptosomal-associated protein 25 (SNAP25), Neurexin 1 (NRXN1), Neuroligin 1 (NLG1), Growth-associated protein 43 (GAP43), and the avian homolog of postsynaptic density protein 95 (PSD95; DLG1) in both embryonic cells and postnatal animals subjected to KA-induced excitotoxicity [18,19,53]. More recently, GH treatment after ONC promoted or preserved anterograde axonal transport and growth cone regrowth, although the magnitude of this effect was modest, and its functional significance remains uncertain [17]. Collectively, these findings establish GH as a multifunctional factor in the retina with the capacity to influence survival, synaptic remodeling, and axonal repair, acting through a complex interplay of signaling cascades which remain to be fully understood.

### 3.3. Endogenous GH During Retinal Diseases

Endogenous GH plays a crucial role in retinal development, particularly in axonal guidance and circuit formation during early stages [15]. However, in adults, most research has focused on its role during retinopathies and optic neuropathies, with findings that are often contradictory or context dependent.

In acromegaly, a condition characterized by chronic GH overproduction, multiple ocular structural alterations have been reported. These include changes in corneal thickness, extraocular muscle morphology, RGCL and inner plexiform layer thickness reduction, as well as changes in choroidal vascular organization, and variable optic nerve fiber and optic nerve head alterations [54,55,56,57,58,59,60]. However, these changes are not consistent across all patients, likely reflecting biological variability in the grade of GH excess [56,61]. Interestingly, early clinical observations suggested that GH excess might facilitate open-angle glaucoma, as reported by Greco and colleagues [62] and earlier by Howard and English [63], who described open-angle glaucoma in 39 out of 100 acromegalic patients. Observational data indicate that GH-deficient diabetic patients have a lower prevalence of retinopathy compared with GH-sufficient diabetics, a finding consistent with a contributory role of the somatotropic axis in this pathogenesis [64]. Importantly, intraocular elevations of GH and IGF-1, rather than serum levels alone, have been implicated in pathological neovascularization and the retinal changes characteristic of proliferative diabetic retinopathy (DR), and interventions that reduce GH/IGF-1 activity (e.g., somatostatin analogues, GH receptor antagonists) can diminish retinal neovascularization in experimental models and certain clinical contexts [65].

In contrast, GH deficiency has also been linked to ocular dysfunctions, including elevated intraocular pressure (IOP), the major risk factor for glaucoma, as observed in GH-deficient children [66], contradicting the notion that only GH excess predisposes to glaucoma. GH excess is further implicated in the aggravation of DR, glaucoma, and corneal diseases, often treated with somatostatin (SST) analogs such as octreotide and lanreotide to inhibit GH secretion [67]. Nevertheless, not all studies find a strong link between acromegaly and retinopathies. For example, Füchtbauer [68] reported increased retinal vascular branching in acromegalic patients but found no significant changes in macroscopic vessel morphology nor higher prevalence of DR, suggesting that GH excess alone may not be a sufficient causal factor.

Experimental data further reflect this complexity. In vitro, GH has been shown to increase proliferation of human retinal microvascular endothelial cells, but this effect depends on the presence of serum in the culture medium [69]. On the other hand, GH deficiency has been associated with abnormal optic nerve and optic disc morphology [70,71,72,73,74]. Moreover, GH therapy in GH-deficient or neurologically compromised children has shown potentially restorative effects: for instance, combined GH and melatonin treatment improved hearing and visual fixation in a child with cerebral palsy, with normalization of visual evoked potentials after therapy [75].

In animal models, findings are also mixed. Transgenic mice overexpressing bovine GH (bGH) exhibited no major changes in retinal histology or function, except for a significant increase in axial length and a reduction in the dominant frequency of oscillatory potentials (from 108 Hz in wild type to 100 Hz in bGH mice) [76]. Conversely, GHR knockout mice (GHR −/−) displayed reduced retinal thickness and lower expression of vascularization-related and neurite outgrowth proteins, as shown by proteomic analysis [15].

Taken together, the effects of endogenous GH on the retina and optic nerve appear to be multifactorial and context-dependent, influenced by both systemic and locally produced GH. This is particularly relevant given that GH can cross the blood–brain and blood–retinal barriers [38,39,40]. While some evidence links GH excess to exacerbation of retinopathies, especially glaucoma and DR, other studies fail to confirm this association [68]. Conversely, GH deficiency and GHR disruption consistently point to structural and functional deficits in the visual system, underscoring the importance of basal GH signaling for its normal development and maintenance [70,71,72,73].

## 4. Hypothalamic Releasing Factors in the Retina

### 4.1. Retinal GH Releasing Hormone (GHRH)

It has been reported that GHRH and its receptor (GHRH-R) are expressed in some retinal cells (Figure 1); but their physiological roles and involvement in retinal pathologies still remain poorly characterized [9,10,22,77]. Evidence from patients with congenital isolated GH deficiency caused by homozygous mutations in the GHRH gene indicates only mild effects on retinal microvasculature, optic disc, and optic cup size, with no significant alterations in macular thickness and overall preserved retinal function [78]. These findings suggest that basal GHRH signaling may not be essential for normal retinal development, but it may become more relevant in pathological or stress-related contexts.

Several studies have proposed that GHRH signaling pathway may participate in retinal stress response, inflammation, and neuroprotection [47,79,80]. For example, the administration of MIA-602, a GHRH antagonist, reduced the expression of key pro-inflammatory mediators such as interleukin-1β (IL-1β), TNF-α, and monocyte chemotactic protein-1 (MCP-1) in the iris and ciliary body, thereby decreasing inflammation in ocular tissues [80]. Conversely, agonist stimulation with subcutaneous administration of MIA-409 promoted RGCs survival in an ONC model in rats, and this effect was associated with increased Akt phosphorylation, a pathway linked to neuronal survival, while administration of the antagonist MIA-602 attenuated this effect [47]. Interestingly, in the same study, MIA-409 enhanced microglial activation and potentiated RGC survival induced by lens injury and zymosan, whereas MIA-602 acted primarily as an anti-inflammatory agent. Notably, MIA-409 significantly increased vitreous GH levels, suggesting that part of its neuroprotective effect might be mediated through GH upregulation [47]. These observations highlight a potential crosstalk between GHRH and GH signaling in retinal repair mechanisms and indicate a positive induction of inflammation that results in neural protection.

Additional evidence indicates that GHRH signaling is dynamically regulated under inflammatory and excitotoxic conditions. Both GHRH-R and GHR are upregulated following lipopolysaccharide (LPS)-induced inflammation in the ciliary epithelium and are expressed by infiltrating leukocytes and macrophages [80]. Similarly, GHRH has been found to colocalize with RGCs in chick embryos and, in the QNR/D retinal cell line, GHRH immunoneutralization reduces cell survival [81,82]. Moreover, GHR expression is upregulated after excitotoxic injury in neuroretinal cells, supporting the idea that GHRH/GH signaling acts as part of an emergency response mechanism during retinal damage [53].

Beyond acute injury, GHRH pathways have also been implicated in diabetic retinopathy. In early models of this disease, the agonist MIA-409 demonstrated protective effects by reducing oxidative stress, exerting antioxidant and anti-inflammatory actions, and decreasing vascular permeability [79]. These findings reinforce the notion that GHRH modulation can influence retinal vascular homeostasis under diabetic conditions.

However, the role of GHRH in retinal pathology is not exclusively protective. Excessive activation of GHRH pathways, particularly through the canonical JAK/STAT3 signaling cascade, has been reported to enhance Th17 cell-mediated inflammation, which may contribute to the progression of ocular degenerative and autoimmune diseases [83]. Furthermore, in models of LPS-induced ocular inflammation, there is a transcriptional upregulation of GHRH-R and induction of JAK/STAT3 signaling, suggesting that under certain inflammatory conditions, GHRH may play a pro-inflammatory deleterious role [84].

Taken together, current evidence portrays GHRH as a context-dependent regulator in the retina, with the potential to act either as a neuroprotective and anti-inflammatory factor during injury and early DR, or as a promoter of inflammation and tissue damage when excessively activated. The dual actions of GHRH and its interaction with GH signaling suggest that the GHRH–GH axis may represent a dynamic adaptive system in retinal homeostasis, with therapeutic potential that requires precise temporal and dose-dependent modulation.

### 4.2. Retinal Ghrelin

Ghrelin was initially identified as a gastric peptide hormone involved in the regulation of feeding behavior and energy homeostasis, but it is now recognized as a ubiquitous signaling molecule with diverse local and systemic actions, including a potent ability to stimulate GH release [85,86,87]. Beyond its endocrine role, ghrelin exerts multiple paracrine and autocrine effects in the CNS and the eye [88,89,90]. In the retina, ghrelin is expressed in Müller glial cells, retinal neurons, and vascular cells [22,88,89]. Its strongest expression, however, has been reported in the ciliary processes of the ciliary body and the epithelium of the iris [89,91], where it is believed to influence smooth muscle contractility and ocular hydrodynamics. Given that GH is expressed in the retina before the hypothalamic–hypophyseal axis becomes functional, ghrelin may act as a local regulator of GH during development, contributing to axonal guidance and maturation of visual circuits [24,92].

The biological actions of ghrelin are primarily mediated by the growth hormone secretagogue receptor type 1a (GHSR-1a), a G-protein coupled receptor that activates multiple intracellular cascades. These include the phospholipase C (PLC) pathway, AMP-activated protein kinase (AMPK), MAPKs, and the PI3K/Akt pathway [93,94,95,96]. Through these mechanisms, ghrelin has been implicated in angiogenesis, neuroprotection, survival, synaptic modulation, and autophagy [97,98,99]. Interestingly, the most abundant isoform in circulation, non-acylated ghrelin, represents nearly 90% of total ghrelin and does not bind GHSR-1a. Nevertheless, this isoform also displays biological effects, raising the possibility of additional, unidentified receptors mediating its functions [100,101].

In ocular pathophysiology, ghrelin has emerged as a potential neuroprotective and antioxidant factor. Systemic administration of ghrelin has been shown to protect against RGC loss in experimental models of glaucoma and DR [98,99,102]. Its relatively small size (28 amino acids) likely facilitates its permeability across the blood–brain and blood–retinal barriers, enabling central and ocular effects [103]. Supporting a clinical association, significantly lower levels of ghrelin have been detected in the aqueous humor of glaucoma patients compared to controls [104]. Since ghrelin also influences the contractile properties of the ciliary body and iris, thereby modulating aqueous humor production and drainage, it has been hypothesized that ghrelin dysregulation could contribute to altered IOP dynamics, an essential factor in glaucoma pathogenesis [93].

Despite its demonstrated protective roles in retinal models and its established role as a GH secretagogue, there is currently no direct experimental or clinical evidence linking retinal or systemic ghrelin to the regulation of retinal GH expression or GH-mediated neuroprotection. Moreover, the strong dependence of ghrelin secretion on nutritional status, being upregulated during fasting and downregulated after feeding, adds complexity to studying its precise roles in ocular physiology. This underscores the need for targeted studies to determine whether ghrelin functions mainly as an independent neuroprotective factor, as a metabolic modulator of retinal physiology, or as an upstream regulator of the somatotropic axis in the eye.

### 4.3. Retinal Thyrotropin Releasing Hormone (TRH)

In addition to its role in regulating the thyroid axis, TRH is well established as a hypothalamic neuropeptide capable of stimulating GH release from pituitary somatotrophs in several vertebrates [105,106,107]. However, TRH expression is not restricted to the hypothalamus, as it has also been found in tissues of the immune, reproductive, and nervous systems, although its precise functions in these contexts remain poorly understood [108,109,110,111]. Within the nervous system, extrahypothalamic expression of TRH and its receptor has been demonstrated in several structures, including areas of the brain involved in neuromodulation and sensory processing, as well as in ocular tissues of fishes, reptiles, birds, and mammals [77,106,112,113,114,115,116,117]. In the retina, a high density of TRH receptors was reported by Sharif and Burt [118] using radiolabeled TRH. Functional studies further demonstrated that TRH exerts differential effects on ON- and OFF-center retinal neurons, suggesting a modulatory role during light adaptation [119]. Consistent with this, retinal TRH levels appear to be influenced by environmental lighting, with higher expression during the day. Moreover, TRH can suppress light-evoked dopamine release and modulate dopamine output from amacrine cells through presynaptic receptors, indicating a possible role in photoreceptive processing and circadian regulation [120,121].

The regulation of retinal TRH release involves complex interactions with other neurotransmitters. Acetylcholine stimulates its release, while somatostatin, GABA, dopamine, and serotonin act as inhibitors [122,123,124,125]. Notably, somatostatin inhibits TRH release in a dose-dependent manner in rat retina in vitro, while TRH in turn inhibits somatostatin signaling [124]. These reciprocal inhibitory interactions are relevant since TRH has been shown to modulate retinal GH release [81], and the suppression of somatostatin may represent one of the mechanisms through which TRH influences local GH actions in the retina.

At present, there is no direct evidence linking TRH to retinal degeneration or retinopathies, either as a deleterious or protective factor. However, its modulatory effects on dopaminergic and cholinergic neurotransmission, as well as its regulatory influence over somatostatin and GH pathways, indicate that TRH may contribute to the fine-tuning of retinal physiology [122,123,124]. Although the exact functional consequences remain unclear, TRH emerges as a candidate molecule of interest for future studies on neuroendocrine regulation of retinal function and GH related neurotrophic processes.

### 4.4. Retinal Gonadotropin Releasing Hormone (GnRH)

The influence of GnRH on pituitary GH expression and release was initially thought to be species-specific and largely restricted to teleost fishes, as increases in circulating GH in response to GnRH administration had been documented mainly in goldfish, grass carp, and common carp; however, more recent evidence indicates that GnRH can also promote GH expression and secretion in the pituitary of non-mammalian amniotes [105,126,127]. In mammals, however, there is no evidence that GnRH directly regulates GH or IGF-1 production. Instead, local GnRH in mammals has been associated with the regulation of growth and differentiation during early embryonic development, particularly before implantation and throughout organogenesis [128]. These developmental roles often occur together with other locally produced hormones, including releasing factors and peptides related to the somatotropic axis, suggesting a context dependent role for GnRH as a paracrine or autocrine modulator.

Clinically, GnRH analogs have sometimes been used as adjunct therapies to enhance GH mediated growth in children with idiopathic GH deficiency, and some reports suggest they may improve growth outcomes in short non-GH deficient children [129,130]. However, the specific mechanistic interactions between GnRH and GH signaling pathways have not been systematically explored. Recent experimental data indicate that combined administration of GH and GnRH does not synergize to enhance neuroprotective or regenerative effects. On the contrary, both hormones appear to partially counteract each other’s actions in models of neural injury [31,32,131].

Regarding the visual system, a limited number of studies have documented GnRH and its receptor expression in both retinal tissue and visual processing areas of the brain. Most of these findings derive from fish models, where GnRH expression has been linked to neuromodulatory functions, although its precise roles in the retina remain undefined [132,133,134,135].

Current data suggest that GnRH itself is unlikely to be a major effector of GH’s neuroprotective actions in the nervous system, especially given the evidence that their combined administration dampens neuroprotective outcomes [31,32,131]. Nevertheless, the observation that many retinopathies show sex-related prevalence or progression patterns raises the possibility that gonadal hormones and potentially GnRH through its systemic and local actions could influence the susceptibility or development of certain retinal disorders [136]. Further studies are needed to determine whether local GnRH signaling contributes directly to retinal physiology or pathology, and whether it interacts with GH or other elements of the somatotropic axis in this context.

### 4.5. Retinal Somatostatin (SST)

SST is classically recognized as the main inhibitor of pituitary GH release [137]. Interestingly, stimulatory actions of SST on GH secretion have also been reported under specific contexts highlighting its context-dependent roles [137,138]. Evidence demonstrates the presence of SST and its receptors in several retinal cell types, including photoreceptors, bipolar cells, amacrine cells, retinal ganglion cells, and Müller glia, in species such as frog, rat, guinea pig, mink, monkey and human [9,10,139,140,141]. Importantly, SST expression occurs across developmental stages, from embryonic life through adulthood, but with significant species-specific differences in its spatial and temporal expression patterns [142,143,144,145].

SST bioactivity in the retina has been studied intensively due to its potential clinical applications, particularly in DR and ischemic retinal disorders [146,147,148]. A growing body of evidence indicates that SST protects neuroretinal cells and the retinal pigment epithelium against excitotoxic damage, oxidative stress, and hyperglycemia-induced injury, as well as in DR models. These effects appear to be at least partly mediated by the modulation of microglial activity and show remarkable diversity depending on the specific receptor subtype activated [149,150,151,152,153,154]. Receptor-specific mechanisms are particularly relevant. Activation of sst2 and sst5 receptors by analogs has been shown to protect RGCs from apoptosis and oxidative stress in experimental models of DR and glaucoma [146,155]. However, the literature presents some contradictions. As reviewed by Fang and colleagues [146], low endogenous levels of SST during early DR correlate with worse neurodegenerative outcomes, while exogenous supplementation of SST or its analogs at later stages exerts protective effects by reducing both neuronal loss and angiogenesis progression. These apparently paradoxical findings may reflect the complex spatiotemporal regulation of SST signaling and receptor subtype involvement.

Although many of SST’s protective effects in DR and glaucoma models appear to be independent of GH and IGF-1 pathways, systemic SST analogs can also influence the GH/IGF-1 axis. In some subgroups of DR patients, treatment with SST analogs has been associated with decreased IGF-1-induced VEGF production, which in turn limits pathological neovascularization [156,157]. STT has dual actions, is directly protective on retinal neurons and indirectly anti-angiogenic via IGF-1 modulation.

Despite these advances, no direct functional connection has yet been established between locally produced retinal SST and retinal GH. This contrasts with the pituitary, where their relationship is well characterized [138]. The retina expresses multiple SST receptor subtypes (sst1–sst5), and this diversity complicates the dissection of specific receptor-mediated actions [9,10,139,140,141]. The development of selective SST analogs and receptor-targeted therapies may provide important insights into the role of SST in retinal physiology and pathology. SST in the retina is as a multifunctional modulator, involved in neurotransmission, neuroprotection, and vascular regulation. While its role as a local regulator of GH in the retina remains unclear, the parallels with CNS functions and the receptor diversity strongly suggest a broader regulatory capacity that warrants further investigation.

## 5. Insulin-like Growth Factor 1 (IGF-1)

In many tissues, IGF-1 is recognized as the classical downstream mediator of pituitary GH effects. However, IGF-1 actions extend beyond its dependence on GH, and the linear GH–IGF-1 endocrine axis that characterizes somatic growth does not fully represent the dynamics of these molecules in the adult nervous system [27]. During development, IGF-1 plays essential roles in the formation and maturation of multiple tissues, including the nervous system [158]. Importantly, even at early developmental stages, IGF-1 expression is at least partially independent of GH, as IGF-1 appears in several tissues before pituitary GH expression begins [159].

Substantial evidence indicates that IGF-1 is crucial for retinal development. Inhibition of IGF-1 receptor signaling impairs the formation of optic vesicles and optic cups derived from human embryonic stem cells, whereas exogenous IGF-1 promotes the formation of laminated retinal tissue containing multiple differentiated retinal cell phenotypes [160]. Additional developmental evidence comes from mice lacking insulin receptor substrate-2, a critical component of IGF-1 intracellular signaling; these animals display great photoreceptor loss by 16 months of age [161], emphasizing the long-term importance of IGF-1 signaling for photoreceptor maintenance.

IGF-1 promotes proliferation, survival, and differentiation of several retinal cell types and exerts neuroprotective actions across diverse models of retinal disease and injury, including ischemia, DR, and retinitis pigmentosa [162,163,164,165,166,167,168]. Mechanistically, IGF-1 activates PI3K/Akt, MAPK/ERK, and anti-apoptotic signaling pathways that are well characterized in both the retina and the brain, where IGF-1 is broadly recognized as a neurotrophic factor [50]. Despite these neuroprotective effects, the outcomes of IGF-1 signaling are highly dose and context dependent. For example, although IGF-1 enhances neuroprotection when administered together with dopamine in a rat model of DR, chronic overexpression of IGF-1 in the retina induces blood–retinal barrier (BRB) breakdown and increases vascular permeability [162,169]. Notably, this disruptive effect is specific to retinal IGF-1 overexpression, since elevated systemic IGF-1 alone does not induce BRB breakdown [169], suggesting that local versus systemic IGF-1 exert distinct biological actions in the retina. Of note, IGF-1 is capable of crossing the blood–brain barrier [170], but this permeability does not necessarily imply functional equivalence between systemic and intraocular IGF-1.

The dual opposite protective and deleterious effects of IGF-1 likely reflect differences in concentration, timing, receptor availability, and interplay with local inflammatory or metabolic factors [162,163]. In the retina, as in the CNS, IGF-1 may act not only directly on neurons and glia but also indirectly through modulation of microglial activation, oxidative stress responses, and vascular homeostasis, especially since its receptor is notably expressed in different cell types (Figure 1) [163,166,168].

Given its pronounced neurotrophic properties, it is reasonable to hypothesize that IGF-1 contributes to GH-mediated neuroprotection (Figure 3). Experimental evidence supports this idea. Martínez-Moreno and colleagues [41] showed that siRNA knockdown of GH or IGF-1, as well as immunoneutralization of IGF-1, reduced QNR/D cell viability; these cells overexpressed rcGH, which elevated IGF-1 levels. Likewise, intravitreal injection of cGH siRNA in developing chicken embryos increased apoptosis in the retina while simultaneously reducing both GH and IGF-1 mRNA levels [171]. Additionally, systemic administration of neuroprotective GH doses in an optic nerve crush model in rats upregulates IGF-1 mRNA levels in the retina during early treatment phases, although local IGF-1 expression decreases after 28 doses despite systemic levels remaining elevated [17]. Together, these observations suggest that IGF-1 contributes, at least in part, to GH’s neuroprotective actions in the retina, and likely in other neural tissues. Nevertheless, the regulation of GH and IGF-1 within the nervous system is more complex than the classical endocrine model implies. Timing, local concentrations, and paracrine/autocrine signaling profoundly influence the balance between neurotrophic and potentially deleterious outcomes. Thus, while the GH–IGF-1 axis clearly interacts in retinal neuroprotection, its regulation in the retina and presumably in the rest of the CNS does not always mirror the canonical endocrine pathway observed in peripheral GH target tissues.

## 6. Other Pituitary Hormones

Besides GH, the anterior pituitary produces and secretes several hormones essential for regulating growth, metabolism, reproduction, stress responses, and sexual development, among other important body functions. These include the follicle-stimulating hormone (FSH), luteinizing hormone (LH), adrenocorticotropic hormone (ACTH), thyroid-stimulating hormone (TSH), and prolactin (PRL), the latter sharing a structural ancestral origin with GH derived from gene duplication events [172,173]. Although the systemic functions of these hormones are generally well characterized, their actions in the CNS and particularly in the retina are less understood. Among them, PRL has gained significant attention for its potential therapeutic effects in retinal degeneration and injury, especially on DR [174,175], while others show sparse or emerging evidence of retinal involvement.

LH receptor (LHR) expression has been detected in the neural retina, predominantly in cone photoreceptors [176]. In agreement with this distribution, adult LHR knockout mice exhibit reduced a-wave and b-wave amplitudes in full-field ERG at high stimulus intensities, reinforcing a functional role in cone physiology [177]. Interestingly, LH levels are elevated in the vitreous humor of diabetic patients with proliferative DR, and its levels in healthy bovine and porcine vitreous, correlate with VEGF abundance, suggesting that LH may interact with angiogenic pathways in pathological contexts [177,178]. However, beyond these associations, LH has no demonstrated roles in retinal neurodegeneration or broader retinopathies, and its interactions with other pituitary hormones, including GH or IGF-1, remain unexplored.

Information regarding ACTH actions in the retina is also scarce. The only report suggesting a neuroprotective effect showed that two weeks of repository corticotropin injections improved visual acuity in patients with acute demyelinating optic neuritis [179]. Structural changes included thinning of the papillomacular bundle, ganglion cell layer, inner plexiform layer, and retinal nerve fiber layer, accompanied by thickening of the inner nuclear, outer plexiform, outer nuclear, and photoreceptor layers [179]. Beyond this isolated clinical observation, ACTH’s retinal actions have been studied mostly in the context of ocular inflammation (reviewed by Crane and colleagues [180]), and its interactions with the somatotropic axis remain unknown.

While thyroid hormones have well established roles in retinal development, particularly during differentiation and maintenance of cone subtypes in mammals, and thyroid autoimmunity can lead to thyroid-associated ophthalmopathy—a severe inflammatory disease with impact on orbital tissues [181,182,183], the specific roles of TSH in the retina have not been investigated. To date, neither TSH nor its receptor has been functionally characterized in retinal cells, and potential interactions with local GH, IGF-1, or other pituitary-derived hormones remain unexplored. A similar situation applies to FSH, for which no expression, receptor distribution, or functional reports exist in the retina, leaving its relevance to retinal physiology or disease unknown.

In contrast to the poorly characterized retinal effects of these hormones, PRL has been extensively studied in the visual system due to its neuroprotective, antioxidant, anti-inflammatory, and angiogenic (or antiangiogenic when cleaved to vasoinhibin) properties [175,184,185,186]. PRL expression has been detected in photoreceptor outer segments, Müller cells, interneurons, ganglion cells, and astrocytes, while its receptor (PRLR) localizes to the photoreceptor nuclear layer, inner nuclear layer, and ganglion cell layer in rat and monkey retinas [187]. Expression in all retinal lineages of fetal baboon retina further supports a developmental regulatory role [188].

PRL is recognized as a potent neurotrophic factor [174]. Its neuroprotective effects have been demonstrated both in vitro and in vivo, including protection against excitotoxicity, ischemic injury, and phototoxicity [189,190,191,192]. Lack of PRL signaling in aged mice correlates with photosensitive retinal dysfunction, photoreceptor apoptosis, microglial activation, and increased expression of pro-apoptotic markers [193]. Several PRL-induced intracellular mechanisms overlap with those activated by GH, including modulation of neurotrophins, regulation of Bcl-2 family proteins, glial involvement, and activation of Akt, ERK1/2, and NF-κB signaling [190,193,194,195]. This overlap is particularly relevant given evidence that GHR and PRLR can form heteromultimers and that GH can bind and activate both receptors under specific conditions [196,197,198]. These findings raise the possibility that some effects attributed to GH in the retina may involve PRLR-mediated mechanisms, and vice versa.

Proteolytic cleavage of PRL by cathepsin-D or matrix metalloproteases generates the vasoinhibin (Vi), a N-terminal fragment with strong antiangiogenic and vasoconstrictive properties [175]. Both the angiogenic effects of full-length PRL and the antiangiogenic actions of vasoinhibin have placed this hormone system at the center of DR research. These molecules are now considered significant regulators of retinal vascular pathology and promising therapeutic tools [186,199]. The vasoinhibin could be a valuable complementary agent in combination therapies involving GH, particularly because one concern associated with GH treatment is its potential to induce or exacerbate angiogenesis [200,201]. Furthermore, in line with the well-characterized proteolytic processing of PRL, there is experimental evidence demonstrating that GH can also undergo proteolytic cleavage, generating bioactive fragments with distinct biological properties [24,202,203]. In avian species, GH undergoes proteolytic processing that generates bioactive N-terminal fragments, including a well-characterized 15 kDa variant produced by cleavage at Arg133–Gly134 [203]. This fragment displays distinct biological activities and, importantly, has been shown to exert neuroprotective effects by enhancing cell survival and inhibiting apoptosis through PI3K/Akt signaling under hypoxic–ischemic conditions [202]. In the embryonic chick retina, full-length GH mRNA identical to the pituitary transcript is locally expressed; however, GH immunoreactivity is predominantly associated with submonomeric 15–16 kDa proteins rather than the canonical 22–25 kDa monomer [24]. These findings support the notion that GH-derived fragments may contribute to GH actions in the nervous system and raise the possibility that similar processing mechanisms could operate in the retina and other CNS regions. Exploring these potential interactions and synergistic mechanisms may provide new insights into the therapeutic potential of both hormones in retinal disease and broader neurodegenerative conditions.

## 7. Other Retinal Growth and Neurotrophic Factors

Possibly, some of the most widely tested molecules for neuroprotection in the retina and the central nervous system are those belonging to the neurotrophin family, including BDNF, neurotrophin-3 (NT-3), NGF, and neurotrophin-4/5 (NT-4/5). Other neurotrophic factors, such as CNTF and the glial cell line–derived neurotrophic factor (GDNF), have also been explored for their capacity to promote survival of retinal cells under various injury conditions (previously reviewed [48,204,205]). According to the U.S. National Library of Medicine clinical trials database, CNTF and NGF remain under active clinical investigation, although none have yet been approved for clinical use.

GH neuroprotective effects in the retina and in other regions of the CNS frequently involve the upregulation of several of these neurotrophic molecules. For example, during hypoxic–ischemic injury GH treatment significantly increases BDNF and NT-3 mRNA expression in the embryonic chicken cerebral pallium [206]. In chicken neuroretinal cells exposed to kainic acid, GH overexpression also upregulates these neurotrophins [18]. Similarly, in an excitotoxic damage model in the chicken retina, chronic intravitreal GH administration elevates BDNF mRNA levels [19]. GH also influences the expression of NGF, GDNF and CNTF in the retina as well as NT-3 presence in the retinal ganglion cell layer after optic nerve crush injury in rats [17]. Notably, several studies indicate that the induction of neurotrophic factor expression requires the presence of cellular injury, as GH treatment alone does not significantly alter BDNF or NT-3 levels under basal conditions [19,29]. This injury-dependence mirrors patterns observed in other neuroprotective paradigms, suggesting that GH may cooperate with damage-activated intracellular programs rather than acting as a constitutive inducer of neurotrophins.

GH has also been shown to modulate the expression of other retinal growth factors. Thus, IGF-1 as previously discussed, bone morphogenetic protein-4 (BMP4), and fibroblast growth factor-2 (FGF2) all respond to GH treatment in the retina [19,41]. Each of these factors independently exerts neuroprotective effects in retinal tissue, although FGF2 appears to promote cell survival without preserving visual function, as assessed by full-field ERG recordings [166,207,208]. It is important to note, however, that GH-induced upregulation of FGF2 has this far been demonstrated only in avian models, and although GH also increases BMP4 expression in the chicken retina, this effect was not observed in rats [17,19,41].

In the context of DR, IGF-1 has long been proposed to play an important role in driving cell and vessel growth, partly through triggering the production of FGFs, vascular endothelial growth factor (VEGF), and platelet-derived growth factor (PDGF) [209]. Yet the relationship between the somatotropic axis and retinal growth factors in DR is not fully understood. On one hand, components of this axis have been implicated in pathological angiogenesis; on the other, GH expression is downregulated in the retina of diabetic rats and humans, and GHRH treatment both restores GH expression and exerts neuroprotective effects in a DR model in rats, observations that complicate the conventional view of GH/IGF-1 as uniformly pro-angiogenic in the retina [80,209,210].

Given these contrasting findings, the involvement of somatotropic-axis-related growth factors in proliferative retinopathies remains unresolved. However, they cannot be dismissed as potential contributors to the outcomes of GH based interventions. As with many neurotrophic factors, their actions may be beneficial or detrimental depending on species, developmental stage, injury type, and the timing and dosage of treatment.

Finally, additional regulatory molecules with established roles in retinal homeostasis and vascular control such as the pigment epithelium-derived factor (PEDF) and norrin, may also intersect with GH signaling pathways, although their participation has not yet been explored [48]. Their pleiotropic effects suggest that broader interactions between GH and multiple trophic networks are likely and warrant further investigation.

## 8. Conclusions

GH has emerged as a potent neuroprotective factor with promising therapeutic potential in the retina. Its multilayered regulation, involving local production, extrapituitary modulation, and interactions with hypothalamic releasing factors, suggests that GH operates within a complex network rather than a simple linear endocrine axis. This complexity, together with GH’s ability to modulate multiple neurotrophic, angiogenic, and survival-related molecules, highlights the possibility of developing combinatory therapies that leverage synergistic pathways. However, important knowledge gaps remain, including the need to define optimal dosing regimens, treatment windows, and species-specific differences, especially since many robust effects have been reported in non-mammalian vertebrates. Establishing the conditions under which GH and its related axis elements (such as IGF-1, GHRH, TRH, ghrelin, SST, PRL or Vi) can replicate their neuroprotective, synaptogenic, and metabolic actions in mammalian retina will be crucial. Overall, the broad influence of the somatotropic axis on retinal resilience positions it as a compelling target for future neuroprotective strategies, with combinatory or multi-target approaches representing a promising direction for therapeutic development.

## Data Availability

No new data were created or analyzed in this study. Data sharing is not applicable to this article.

## Supplementary Materials

The following supporting information can be downloaded at: https://www.mdpi.com/article/10.3390/cimb48030296/s1.
