# Supplementary material for: Role of Growth Hormone (GH) and Other Somatotropic Axis Elements in Retinal Neuroprotection
Source: Curr Issues Mol Biol. 2026 Mar 11;48(3):296. doi: 10.3390/cimb48030296 (PMC13024951; doi:10.3390/cimb48030296)
Supplement: Supplementary file 1 [file cimb-48-00296-s001.zip › Code.rtf]

# genes_major_grouped_clustermap_and_violins_FIXED2_3orders_SAFE.pyimport scanpy as scimport pandas as pdimport numpy as npimport seaborn as snsimport matplotlib.pyplot as pltimport anndata as adimport scipy.sparse as spfrom tqdm import tqdmfrom mpl_toolkits.axes_grid1 import make_axes_locatable# ===== Files =====mtx_file = "GSE199317_ONC-retina.mtx"cell_file = "GSE199317_ONC-retina_cell.tsv"gene_file = "GSE199317_ONC-retina_gene.tsv"celltype_file = "GSE199317_ONC-retina_celltype.tsv"print("Loading data...")adata = sc.read_mtx(mtx_file).Tgenes = pd.read_csv(gene_file, header=None, sep="\t", dtype=str)cells = pd.read_csv(cell_file, header=None, sep="\t", dtype=str)celltypes = pd.read_csv(celltype_file, sep="\t", header=0, dtype=str)# Setup namesadata.var_names = genes[0].astype(str)adata.var_names_make_unique()adata.obs_names = cells[0].astype(str)# Only use major annotations (ignore granular)celltypes = celltypes.set_index("cell")adata.obs["cell_type_major_orig"] = adata.obs_names.map(celltypes["cell_type_major"])# Keep only cells that have a major annotationadata = adata[~adata.obs["cell_type_major_orig"].isna()].copy()print(f"??Working with {adata.n_obs} cells (with major annotation)")# Keep raw and create normalized log1p (standard for scRNA-seq figures)adata.raw = adata.copy()sc.pp.normalize_total(adata, target_sum=1e4)sc.pp.log1p(adata)# ===== Genes of interest =====somatotropic_genes = ["Gh", "Ghr", "Ghrh", "Ghrhr", "Ghrl", "Ghsr",                      "Trh", "Trhr", "Gnrh1", "Gnrhr",                      "Sst", "Sstr1", "Sstr2", "Sstr3", "Sstr4", "Sstr5",                      "Igf1", "Igf1r"]housekeeping_genes = ["Gapdh", "Actb", "Rps18"]  # Rpl13a/Hprt removedgenes_interest = somatotropic_genes + housekeeping_genespresent_genes = [g for g in genes_interest if g in adata.var_names]missing_genes = [g for g in genes_interest if g not in adata.var_names]print("Genes present:", present_genes)if missing_genes:    print("Missing genes (not found in var_names):", missing_genes)# helper to flatten sparse/densedef flatten_vals(x):    if sp.issparse(x):        return np.asarray(x.todense()).ravel()    return np.asarray(x).ravel()# ===== Define grouping rules (priority order) =====target_groups = [    "Muller glia",    "Muller glia IFN",    "Astrocyte",    "Microglia",    "Cycling microglia",    "Neutrophil",    "Monocyte/DC",    "Ear2+ monocyte",    "Monocyte/macrophage",    "B cell",    "Plasma B cell",    "T cell",    "Endothelial",    "Pericyte",    "RGC",    "Amacrine cell",    "Bipolar cell",    "Horizontal cell",    "Photoreceptor"]def map_celltype_to_target(orig):    if pd.isna(orig):        return None    o = orig.lower()    if "ifn" in o and "muller" in o:        return "Muller glia IFN"    if "muller" in o:        return "Muller glia"    if "microglia" in o and "cycling" in o:        return "Cycling microglia"    if "microglia" in o:        return "Microglia"    if "astro" in o:        return "Astrocyte"    if "neutro" in o:        return "Neutrophil"    if "ear2" in o or "ear-2" in o or "ear 2" in o:        return "Ear2+ monocyte"    if "mon/dc" in o or "monocyte/dc" in o or ("dc" in o and "mon" in o):        return "Monocyte/DC"    if "mon/mac" in o or "monocyte/mac" in o or ("mac" in o and "mon" in o):        return "Monocyte/macrophage"    if "monocyte" in o or "mon " in o or o.startswith("mon"):        return "Monocyte/macrophage"    if "b cell" in o or o.startswith("b cell") or ("plasma" not in o and "b " in o and "pl" not in o):        return "B cell"    if "plasma" in o:        return "Plasma B cell"    if "t cell" in o or o.startswith("t cell") or "cd4" in o:        return "T cell"    if "endothel" in o:        return "Endothelial"    if "pericy" in o:        return "Pericyte"    if "rgc" in o or "retinal ganglion" in o or "retina ganglion" in o:        return "RGC"    if "amacrine" in o:        return "Amacrine cell"    if "bipolar" in o:        return "Bipolar cell"    if "horizontal" in o:        return "Horizontal cell"    if "photoreceptor" in o or "rod" in o or "cone" in o:        return "Photoreceptor"    if "low umi" in o or "low_umi" in o:        if "rgc" in o:            return "RGC"        if "microglia" in o:            return "Microglia"        if "muller" in o:            return "Muller glia"    return None# Apply mapping and produce mapping table for Excelorig_unique = sorted(adata.obs["cell_type_major_orig"].unique())mapping_rows = []adata.obs["cell_type_major"] = adata.obs["cell_type_major_orig"].map(map_celltype_to_target)for orig in orig_unique:    mapped = map_celltype_to_target(orig)    mapping_rows.append({"original": orig, "mapped_to": mapped if mapped is not None else "DROP"})mapping_df = pd.DataFrame(mapping_rows)# Keep only cells that were mapped to one of the target groupsadata = adata[~adata.obs["cell_type_major"].isna()].copy()print(f"??After grouping: {adata.n_obs} cells assigned to {adata.obs['cell_type_major'].nunique()} groups")# ===== Compute metrics per grouped celltype x gene =====rows = []groups_sorted = sorted(adata.obs["cell_type_major"].unique(), key=lambda x: target_groups.index(x) if x in target_groups else 999)for grp in tqdm(groups_sorted, desc="Groups"):    idx = adata.obs.index[adata.obs["cell_type_major"] == grp].tolist()    n_cells = len(idx)    if n_cells == 0:        continue    for gene in present_genes:        vals_raw = flatten_vals(adata.raw[idx, gene].X)  # counts (raw)        vals_norm = flatten_vals(adata[idx, gene].X)     # normalized log1p        positives_mask = vals_raw > 0        positives = int(np.count_nonzero(positives_mask))        porcentaje = (positives / n_cells) * 100.0 if n_cells > 0 else 0.0        # compute positive-only statistics using the normalized values but only where raw counts > 0        if positives > 0:            media_positiva = float(np.nanmean(vals_norm[positives_mask]))            mediana_positiva = float(np.nanmedian(vals_norm[positives_mask]))            std_positiva = float(np.nanstd(vals_norm[positives_mask]))        else:            media_positiva = np.nan            mediana_positiva = np.nan            std_positiva = np.nan        media_general = float(np.nanmean(vals_norm)) if n_cells > 0 else np.nan        mediana_general = float(np.nanmedian(vals_norm)) if n_cells > 0 else np.nan        std_general = float(np.nanstd(vals_norm)) if n_cells > 0 else np.nan        rows.append({            "gene": gene,            "group": ("Housekeeping" if gene in housekeeping_genes else "Somatotropic"),            "celltype": grp,            "total_celulas": n_cells,            "positivas": positives,            "porcentaje": round(porcentaje, 2),            "media_general": media_general,            "media_positiva": media_positiva,            "mediana_general": mediana_general,            "mediana_positiva": mediana_positiva,            "std_general": std_general,            "std_positiva": std_positiva        })df = pd.DataFrame(rows)# Pivot tablespivot_perc = df.pivot(index="celltype", columns="gene", values="porcentaje").fillna(0)pivot_media_pos = df.pivot(index="celltype", columns="gene", values="media_positiva")# Mask: mean-positive -> NaN cuando % == 0 (para que quede blanco)mask_zero = (pivot_perc <= 0.0)pivot_media_pos_masked = pivot_media_pos.copy().astype(float)pivot_media_pos_masked[mask_zero] = np.nan# Orden para Excel (igual que tu script original)col_order = pivot_media_pos_masked.mean(axis=0, skipna=True).sort_values(ascending=False).index.tolist()row_order = pivot_media_pos_masked.mean(axis=1, skipna=True).sort_values(ascending=False).index.tolist()pivot_perc_excel = pivot_perc.loc[row_order, col_order]pivot_media_pos_masked_excel = pivot_media_pos_masked.loc[row_order, col_order]# ===== Save Excel including mapping sheet (sin cambiar nada más) =====with pd.ExcelWriter("Expression_major_grouped_summary.xlsx", engine="openpyxl") as writer:    df.to_excel(writer, sheet_name="Detailed_summary", index=False)    pivot_perc_excel.to_excel(writer, sheet_name="Percent_positive")    pivot_media_pos_masked_excel.to_excel(writer, sheet_name="Mean_positive_masked")    pivot_media_pos.to_excel(writer, sheet_name="Mean_positive_raw")    mapping_df.to_excel(writer, sheet_name="Grouping_map", index=False)print("??Excel saved: Expression_major_grouped_summary.xlsx")# ===== Orden lógico de tipos celulares (neuronas →glía →otras) =====celltype_order_desired = [    # Neuronas    "RGC", "Amacrine cell", "Bipolar cell", "Horizontal cell", "Photoreceptor",    # Glía    "Muller glia", "Muller glia IFN", "Astrocyte", "Microglia", "Cycling microglia",    # Vasculares / otras    "Endothelial", "Pericyte",    # Inmunes/hematopoyéticas    "T cell", "B cell", "Plasma B cell", "Monocyte/DC", "Ear2+ monocyte",    "Monocyte/macrophage", "Neutrophil"]# Mantener solo las que existen para evitar KeyErrorcell_order = [ct for ct in celltype_order_desired if ct in pivot_media_pos_masked.index]# ===== Función para heatmap con anotaciones (%) y blancos donde %==0 =====def plot_heatmap(data_mean, data_perc, fname):    n_rows, n_cols = data_mean.shape    cell_w = 0.70    cell_h = 0.45    fig_w = max(8, n_cols * cell_w)    fig_h = max(6, n_rows * cell_h)    fig, ax = plt.subplots(figsize=(fig_w, fig_h))    mask = data_mean.isna()    vmin = 0.0    vmax = 1.0 if np.all(np.isnan(data_mean.values)) else max(1.0, float(np.nanmax(data_mean.values)))    cmap = sns.color_palette("YlGnBu", as_cmap=True)    sns.heatmap(        data_mean, ax=ax, cmap=cmap, vmin=vmin, vmax=vmax,        mask=mask, cbar=False, linewidths=0.6, linecolor="white",        xticklabels=True, yticklabels=True, square=False    )    divider = make_axes_locatable(ax)    cax = divider.append_axes("right", size="3%", pad=0.35)    sm = plt.cm.ScalarMappable(cmap=cmap, norm=plt.Normalize(vmin=vmin, vmax=vmax))    sm.set_array([])    cbar = fig.colorbar(sm, cax=cax)    cbar.set_label("Mean expression (log1p normalized,\npositive cells only)", fontsize=9)    cbar.ax.tick_params(labelsize=8)    if max(n_rows, n_cols) <= 20:        annot_fs = 8    elif max(n_rows, n_cols) <= 30:        annot_fs = 7    else:        annot_fs = 6    for i, ct in enumerate(data_mean.index):        for j, gene in enumerate(data_mean.columns):            perc_val = data_perc.iloc[i, j]            bg = data_mean.iloc[i, j]            if np.isnan(bg):                txt_color = "black"            else:                rel = (bg - vmin) / (vmax - vmin + 1e-9)                txt_color = "white" if rel > 0.55 else "black"            ax.text(j + 0.5, i + 0.5, f"{perc_val:.2f}", ha="center", va="center",                    fontsize=annot_fs, fontweight="bold", color=txt_color)    ax.set_xticklabels(data_mean.columns, rotation=90, fontsize=max(8, annot_fs+1))    ax.set_yticklabels(data_mean.index, rotation=0, fontsize=max(9, annot_fs+1))    ax.set_xlabel("")    ax.set_ylabel("")    plt.tight_layout(rect=[0, 0, 0.88, 1])    plt.savefig(fname, dpi=300, bbox_inches="tight")    plt.close()    print(f"??Saved {fname}")# ======= TRES CLUSTERMAPS (solo cambia el orden de genes; filas = orden lógico) =======# 1) Orden MANUAL lógico de genesorder_manual = [    # Housekeeping    "Gapdh", "Actb", "Rps18",    # GH axis    "Gh", "Ghr",    # IGF axis    "Igf1", "Igf1r",    # Hypothalamic releasing hormones and receptors    "Ghrh", "Ghrhr", "Trh", "Trhr", "Gnrh1", "Gnrhr",    # Somatostatin + receptors    "Sst", "Sstr1", "Sstr2", "Sstr3", "Sstr4", "Sstr5",    # Ghrelin axis    "Ghrl", "Ghsr"]order_manual = [g for g in order_manual if g in pivot_media_pos_masked.columns]data_mean_manual = pivot_media_pos_masked.loc[cell_order, order_manual]data_perc_manual = pivot_perc.loc[cell_order, order_manual]plot_heatmap(data_mean_manual, data_perc_manual, "clustermap_major_grouped_order_manual.png")# 2) Orden por EXPRESIÓN PROMEDIO (descendente)order_expression = pivot_media_pos_masked.mean(axis=0, skipna=True).sort_values(ascending=False).index.tolist()data_mean_exp = pivot_media_pos_masked.loc[cell_order, order_expression]data_perc_exp = pivot_perc.loc[cell_order, order_expression]plot_heatmap(data_mean_exp, data_perc_exp, "clustermap_major_grouped_order_expression.png")# 3) Orden por CLUSTERING JERÁRQUICO (dendrograma de genes)# Evitamos NaNs en el clustering (solo para calcular el orden); filas ya fijas en cell_ordercluster_matrix = pivot_media_pos_masked.loc[cell_order, :].fillna(0.0)if cluster_matrix.shape[1] > 1:    cg = sns.clustermap(cluster_matrix, row_cluster=False, col_cluster=True,                        cmap="YlGnBu", vmin=0, vmax=max(1.0, float(cluster_matrix.values.max())),                        yticklabels=False, xticklabels=False, cbar=False)    cluster_order = [cluster_matrix.columns[i] for i in cg.dendrogram_col.reordered_ind]    plt.close(cg.fig)else:    cluster_order = order_expression.copy()data_mean_cluster = pivot_media_pos_masked.loc[cell_order, cluster_order]data_perc_cluster = pivot_perc.loc[cell_order, cluster_order]plot_heatmap(data_mean_cluster, data_perc_cluster, "clustermap_major_grouped_order_clustered.png")# ===== Violin + box plots (idéntico a tu script) =====topN = 6group_counts = adata.obs["cell_type_major"].value_counts()top_groups = group_counts.index[:topN].tolist()print("Top groups (for violins):", top_groups)genes_to_plot = [g for g in housekeeping_genes if g in present_genes]plot_rows = []max_cells_per_group = 2000rng = np.random.default_rng(seed=42)for grp in top_groups:    idx_all = adata.obs.index[adata.obs["cell_type_major"] == grp].tolist()    if len(idx_all) > max_cells_per_group:        idx = list(rng.choice(idx_all, size=max_cells_per_group, replace=False))    else:        idx = idx_all    for gene in genes_to_plot:        vals = flatten_vals(adata[idx, gene].X)        for v in vals:            plot_rows.append({"celltype": grp, "gene": gene, "expr_log1p": v})plot_df = pd.DataFrame(plot_rows)for gene in genes_to_plot:    plt.figure(figsize=(max(6, len(top_groups) * 0.9), 5))    sub = plot_df[plot_df["gene"] == gene]    sns.violinplot(x="celltype", y="expr_log1p", data=sub, order=top_groups,                   inner=None, cut=0, scale="width")    sns.boxplot(x="celltype", y="expr_log1p", data=sub, order=top_groups,                showcaps=True, boxprops={'facecolor': 'none', "linewidth": 1},                showfliers=False, whiskerprops={'linewidth': 1})    plt.title(f"{gene} expression across top {topN} groups (log1p normalized)", fontsize=12)    plt.xlabel("")    plt.ylabel("Expression (log1p normalized)", fontsize=10)    plt.xticks(rotation=45, ha='right')    plt.tight_layout()    fname = f"violin_{gene}_top{topN}.png"    plt.savefig(fname, dpi=300, bbox_inches="tight")    plt.close()    print("Saved", fname)print("All outputs saved: Expression_major_grouped_summary.xlsx, 3 clustermaps (manual/expression/clustered), violin plots.")
